# Feasibility of a home-based computerized cognitive training for pediatric patients with congenital or acquired brain damage: An explorative study

**DOI:** 10.1371/journal.pone.0199001

**Published:** 2018-06-20

**Authors:** Claudia Corti, Geraldina Poggi, Romina Romaniello, Sandra Strazzer, Cosimo Urgesi, Renato Borgatti, Alessandra Bardoni

**Affiliations:** 1 Neuro-oncological and Neuropsychological Rehabilitation Unit, Scientific Institute IRCCS Eugenio Medea, Bosisio Parini, Lecco, Italy; 2 Neuropsychiatry and Neurorehabilitation Unit, Scientific Institute IRCCS Eugenio Medea, Bosisio Parini, Lecco, Italy; 3 Acquired Brain Injury Unit, Scientific Institute IRCCS Eugenio Medea, Bosisio Parini, Lecco, Italy; 4 Laboratory of Cognitive Neuroscience, Department of Languages and Literatures, Communication, Education and Society, University of Udine, Udine, Italy; TNO, NETHERLANDS

## Abstract

**Objectives:**

Pediatric brain damage is associated with various cognitive deficits. Cognitive rehabilitation may prevent and reduce cognitive impairment. In recent years, home-based computerized cognitive training (CCT) has been introduced in clinical practice to increase treatment opportunities for patients (telerehabilitation). However, limited research has been conducted thus far on investigating the effects of remote CCT for the juvenile population in contexts other than English-speaking countries. The aim of the present study was to investigate the feasibility of a home-based CCT in a group of Italian adolescents with brain damage. A commercially available CCT (Lumosity) developed in the English language was used due to the lack of telerehabilitation programs in the Italian language that allow stimulation of multiple cognitive domains and, at the same time, remote automatic collection of data. Thus, this investigation provides information on the possibility of introducing CCT programs available in foreign languages in countries with limited investment in the telerehabilitation field.

**Methods:**

32 adolescents aged 11–16 with a diagnosis of congenital or acquired (either traumatic or non-traumatic) brain damage participated in the study. They received 40 training sessions (5 days/week for 8 weeks). Before starting the training program, they received face-to-face demonstration of training exercises and written instructions in their mother tongue. The feasibility of both training and study design and procedures was assessed through 9 criteria taken from extant literature.

**Results:**

All 9 feasibility criteria were met. 31 out of the 32 participants demonstrated adherence to the training program. 94.2% of training sessions were completed in the recommended timeframe. No significant technical issue was found.

**Conclusions:**

Telerehabilitation seems to be a feasible practice for adolescents with brain damage. A training program developed in a foreign language can be used to counter the unavailability of programs in patients’ mother tongue.

**Trial registration:**

The trial is registered with the ISRCTN registry with study ID ISRCTN59250807

## Introduction

Cognitive deficits are among the most disabling long-term consequences of childhood congenital or acquired brain damage, which reflect insults to the central nervous system in the maturational phase [1–3]. Deficits in global intelligence or single cognitive domains are frequently observed, with possible impact on quality of life [1; 3–4].

Cognitive rehabilitation is considered necessary for patients with brain damage, in order to limit long-term cognitive decay and reduce associated vocational, social and psychological costs [5–8]. The efficacy of rehabilitation treatment increases if programs start early, provide intensive stimulation and continue during the recovery phase at home [9]. Even individuals who do not have general learning difficulties or specific cognitive impairments may benefit from cognitive stimulation, as they can improve their performance level as a result [10–11]. Moreover, it seems that stimulating cognitive domains improves myelination and is associated with increased brain connectivity. Furthermore, after cognitive training, an increase in cortical thickness has also been reported in healthy individuals [12]. Given these considerations, stimulation of cognitive functions during development in patients with brain damage may enhance the functional reorganization of altered neural networks and boost cognitive performance, regardless of the diagnosis (congenital or acquired damage) and the specific cognitive profile of participants.

Traditional cognitive rehabilitation is performed in specialized centers, where face-to-face or group interventions are delivered [13]. However, this type of intervention has limits linked to time, cost and accessibility and may introduce heterogeneity in treatment practices [9; 13–15]. Recently, new rehabilitation programs based on technological devices have been introduced to increase opportunities and the consistency of rehabilitation. The use of technology for rehabilitation also allows for the provision of services remotely and in a non-medical setting [9; 16]. This practice is referred to as telerehabilitation. Telerehabilitation allows care continuity and limits time and economic demands for families and institutes. Moreover, it enables precise monitoring of patients’ performance through online tracking [9].

Studies on the feasibility and efficacy of telerehabilitation programs that aim to stimulate cognitive functions in pediatric patients with brain damage are still limited and have often involved low sample sizes [17–18]. These interventions have been tested in patients with acquired injuries, pointing to promising results [14; 19–30]. No evidence is currently available for children with congenital damage, as studies are still ongoing or haven’t been published yet [31–32]. Furthermore, thus far the interest in telerehabilitation programs for the juvenile population has mainly focused on countries such as the United States, the Netherlands, Australia and Taiwan [33], while limited research has been conducted in other contexts.

In order to extend data on this issue, the present study aimed to investigate the feasibility of home-based computerized cognitive training (CCT) in a group of 11 to 16 year-old Italian adolescents. Participants had either acquired or congenital brain damage. Since no previous study has included a largely mixed population of children with brain damage, it seemed worthwhile to verify whether the challenges associated with experiencing the same training program varied among subgroups of patients with different diagnoses. This may provide useful indications for treatment structuring and provision.

A commercially available CCT (Lumosity) developed in the English language was used, due to the lack of telerehabilitation programs in the Italian language that allow both remote multiple cognitive stimulation and remote automatic collection of data. Indeed, considering the interdependence of different cognitive systems [34–35], multiple stimulation of different cognitive domains may have the greatest impact on cognitive outcome in patients [23].

This study is the preliminary step of a wider research project that aims to evaluate the efficacy of the CCT (the trial is registered with ISRCTN registry with study ID ISRCTN59250807). The investigation of the feasibility of a program within a specific population is of great importance to verify whether the intervention is sustainable and acceptable for participants, before testing its effects [36]. Moreover, it allows the evaluation of whether the research methodology used for the study requires any modifications before extending it to a larger study [36]. To evaluate both feasibility of training and feasibility of the study design and procedures, we used 9 criteria taken from previous research on the feasibility of a similar home-based CCT in adolescents with traumatic brain injuries [23]. These criteria were based on relevant recommendations for conducting research on feasibility [37–38].

An important challenge for this study compared to previous research on the same issue [14; 19–32] is the use of a CCT developed in a foreign language (English) and delivered to Italian participants. For this reason, the findings of this study may provide important knowledge on the possibility of importing CCTs to countries with poor investment in the telerehabilitation field.

While proper testing of the efficacy of the CCT is not the aim of this study, we also provided preliminary data on performance of the CCT and explored whether the CCT effects may be modulated by important demographic and clinical variables, such as patients’ cognitive proficiency, gender and age range.

## Research objectives and hypotheses

We aimed to investigate the feasibility of a home-based CCT in a sample of 11 to 16 year-old Italian patients with acquired or congenital brain damage.

We hypothesized that the training program could appear feasible to participants due to the time-bound (about 20 minutes per day) daily commitment and the pleasantness of the exercises. With respect to the language issue, we hypothesized that the use of simple expedients to overcome the language barrier (such as a precise selection of non-language mediated games, an initial face-to-face demonstration of the exercises and the providing of instructions in patients’ mother tongue) could allow the adoption of a web platform in English to be acceptable for participants.

## Materials and methods

The feasibility study was registered with the ISRCTN registry after the start of patient recruitment, because registration was not required by the study sponsor (Scientific Institute IRCCS Eugenio Medea, Bosisio Parini, Italy). The main study on efficacy was registered (ID number ISRCTN59250807) after the evaluation of feasibility outcomes. The authors confirm that all ongoing and related trials for this intervention are registered. The protocol for this trial and supporting CONSORT checklist are available as Supporting Information; see S1 Protocol and S1 Checklist.

The research project methodology and all related materials were examined and approved by the Ethics Committee of the Scientific Institute IRCCS Eugenio Medea, Bosisio Parini, Italy (#284 Rev. 1; 1 March 2016). All procedures were in agreement with the principles expressed in the Declaration of Helsinki.

Parents of the enrolled participants were asked to provide written informed consent in order to allow data collection and analysis for study purposes. Informed consent was obtained from all participants.

### Inclusion criteria and participant recruitment

Participants were recruited among adolescents with congenital or acquired brain damage who had been referred to the Neurorehabilitation Units of the Scientific Institute IRCCS E. Medea in the year before the research onset. For participants with congenital brain damage, we included individuals with alteration of the brain present at birth due to different causes, such as prenatal or perinatal stroke, cerebral palsy or cerebellar malformation. According to the Brain Injury Association of America (https://www.biausa.org/brain-injury/about-brain-injury/basics/overview), we included in the category of acquired brain damage both injuries of a traumatic nature (e.g., falls, assaults, sports injuries, pedestrian injuries, bicycle/motorcycle crashes) and injuries of a non-traumatic nature (e.g., stroke, infectious disease, brain tumors, lack of oxygen and toxic exposure). Patients with acquired brain damage were considered eligible for the study only if they were in a chronical phase (i.e., at least 1 year post-injury).

For the whole sample, inclusion criteria for eligibility were: age between 11 and 16 years old, as cognitive demands are generally high at this age and individuals are usually able to use technological devices; being native Italian speakers, as demonstrations and instructions on training games were provided in the Italian language. Exclusion criteria were: severe sensory or motor deficits that could not be corrected through compensatory tools and could interfere with training execution and assessment; being simultaneously involved in a different cognitive rehabilitation treatment, to prevent excessive demands on patients and possible interference on training adherence rates; a diagnosis of photosensitive epilepsy, as a computer-based stimulation could produce negative health effects in these patients.

No selection based on intellectual performance was adopted, as the study intended to investigate the feasibility of the training program among the general population of children with brain damage, who display different severity levels and heterogeneous cognitive functioning. Moreover, the main study aims to assess the effects of the selected CCT with respect to different levels of cognitive functioning. Therefore, in this preliminary study on feasibility, subjects with different cognitive profiles were included in order to test the presence of compliance issues that may interfere with training attendance in a specific group of subjects, which could alter data on efficacy in the main study.

Patients’ recruitment was conducted by a research team member who contacted families of eligible participants by phone to propose the project. In case of assent, parents were requested to complete the informed-consent forms related to the project.

Recruitment for this preliminary study on feasibility started on 02/03/2016, after the approval of the research project by the Ethics Committee of Scientific Institute IRCCS E. Medea, Bosisio Parini, Italy (#284 Rev. 1; 1 March 2016). Recruitment ended on 31/08/2016.

### Study design and procedure

This study represents the preliminary phase of a single-center clinical controlled trial (the research is registered with the Italian Ministry of Health Trial, with protocol number 44249 of 08/09/2016 and with ISRCTN registry with study ID ISRCTN59250807; see S1). The clinical trial is expected to be completed within December 2018. The main study applies a stepped-wedge research design, randomly assigning patients to one of 2 groups with different research conditions (see Fig A in S1 Protocol): Group 1 (G1) receives the training program for 8 weeks, followed by a comparable time of no-treatment; Group 2 (G2) remains on the stand-by list for 8 weeks (no-treatment) and then receives the cognitive training program for the following 8 weeks. More specifically, all participants are initially evaluated through a battery of neurocognitive tests tapping all cognitive domains stimulated by the training program and questionnaires on adjustment (T1). Then, they are randomized into two groups. Children of G1 immediately start the 2-month training (step 1) and are re-evaluated at T2 after the training period. Then, they enter a 2-month non-treatment period (step 2). For G2 the two steps are inverted: in step 1 children wait and serve as control, while at step 2 they start the training program. At T3, G2 is evaluated soon after treatment, while G1 receives a short term (2 months) follow-up assessment. 6 months after the end of the treatment, a long-term follow-up assessment is performed for both G1 (T4) and G2 (T5), in order to check for long-lasting treatment effects. The main study is conducted in accordance with CONSORT guidelines for non-pharmacological interventions [39–40]. For the main study, a final sample of 60 patients was set in order to detect within-group change of moderate effect size (Cohen’s *d* = 0.47) [45] with a power of 0.95 and alfa level set at p < 0.05. The software G Power 3 was used for this estimation [41].

Participants of this feasibility study were the ones who accepted to participate in the wider research project in the first 6 months, taking into account an estimated 18-month period of enrollment for the main study. In these 6 months, we succeeded in recruiting 32 participants, of which 42% were assigned to G1 and 58% to G2. This sample size is larger than the sample size of the pilot study we used to define the feasibility outcome measures of this study [23].

In accordance with the main study, participants of this feasibility study received a baseline assessment first. After this test session, they received a face-to-face demonstration of how to carry out the training games and were given written instructions in the Italian language on game rules and objectives. In order to better ensure comprehension of the exercises, during the demonstration session patients were asked to play each exercise under the supervision of a research team member. Then, they were provided with free access to the CCT, receiving a personal username and a password.

During the intervention, participants were asked to complete 40 sessions of the CCT at home: they were expected to be involved in the training program for 20 minutes per working day for 8 weeks (5 days per week for 8 weeks). The order of the games was fixed and identical for all participants. Weekly telephone-based contact with the participants and their parents was conducted by a research team member, with the aim of sustaining training adherence and motivation and recording the reasons of any eventual drop-outs.

Data on training performance were collected on a remote database available to the CCT provider. Number of sessions completed, number of games played per day and the daily result of each exercise was recorded for each patient.

A post-training assessment within a week after the 8-week training period and follow-up assessments according to the evaluation steps set for G1 and G2 in the main study (see Fig A in S1 Protocol) were conducted: participants received the same tests and questionnaires proposed as part of the baseline assessment. At the post-training assessment, they were also asked to complete a questionnaire on training acceptability.

**Fig 1:**
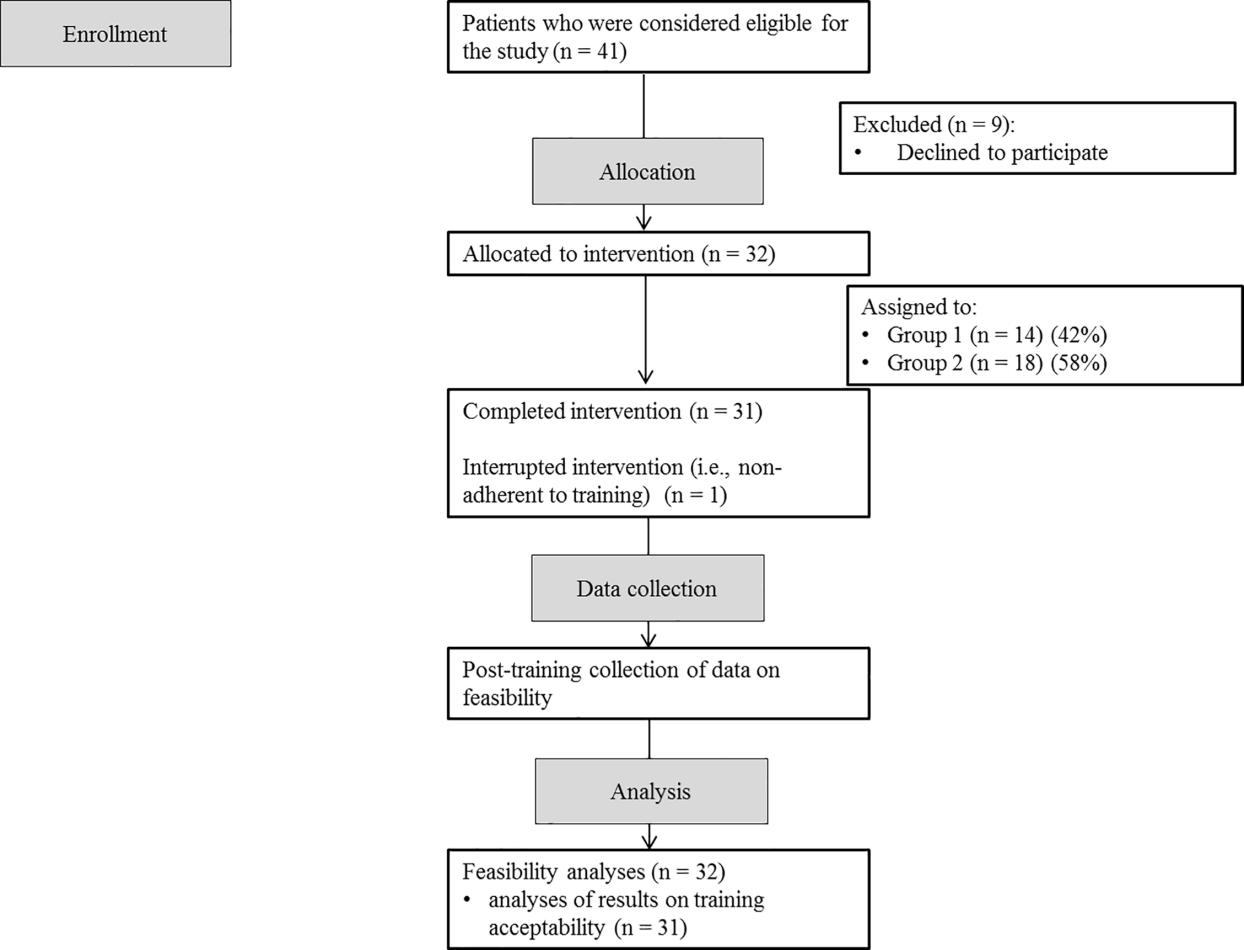
CONSORT flowchart of participant enrollment, inclusion, and involvement.

The flowchart of this feasibility study is presented in Fig 1.

### Intervention

The CCT selected for this study was Lumosity Cognitive Training^TM^ [42], a web-based platform developed in the United States, which provides games that aim to stimulate different cognitive domains. This program is available only in English. However, it was chosen for this study due to the unavailability of an Italian brain-training program that contemporarily focused on a wide array of cognitive functions and allowed precise remote data collection and monitoring. Cognitive domains stimulated by Lumosity^TM^ Cognitive Training include memory, attention, speed, cognitive flexibility and problem-solving. This CCT was selected for other important features considered to be necessary for our study: i) it is adaptive, modifying the complexity of the games based on the individual performance; this aspect is particularly relevant considering that a sample of individuals with brain damage selected without taking into account intellectual ability can be very inhomogeneous with respect to cognitive functioning; ii) it allows for intensive daily training of a limited duration (about 20 minutes), saving patients from excessive cognitive requests at an age where everyday demands are high both at school and at home; iii) it has already been used among different populations, both healthy and clinical.

**Fig 2:**
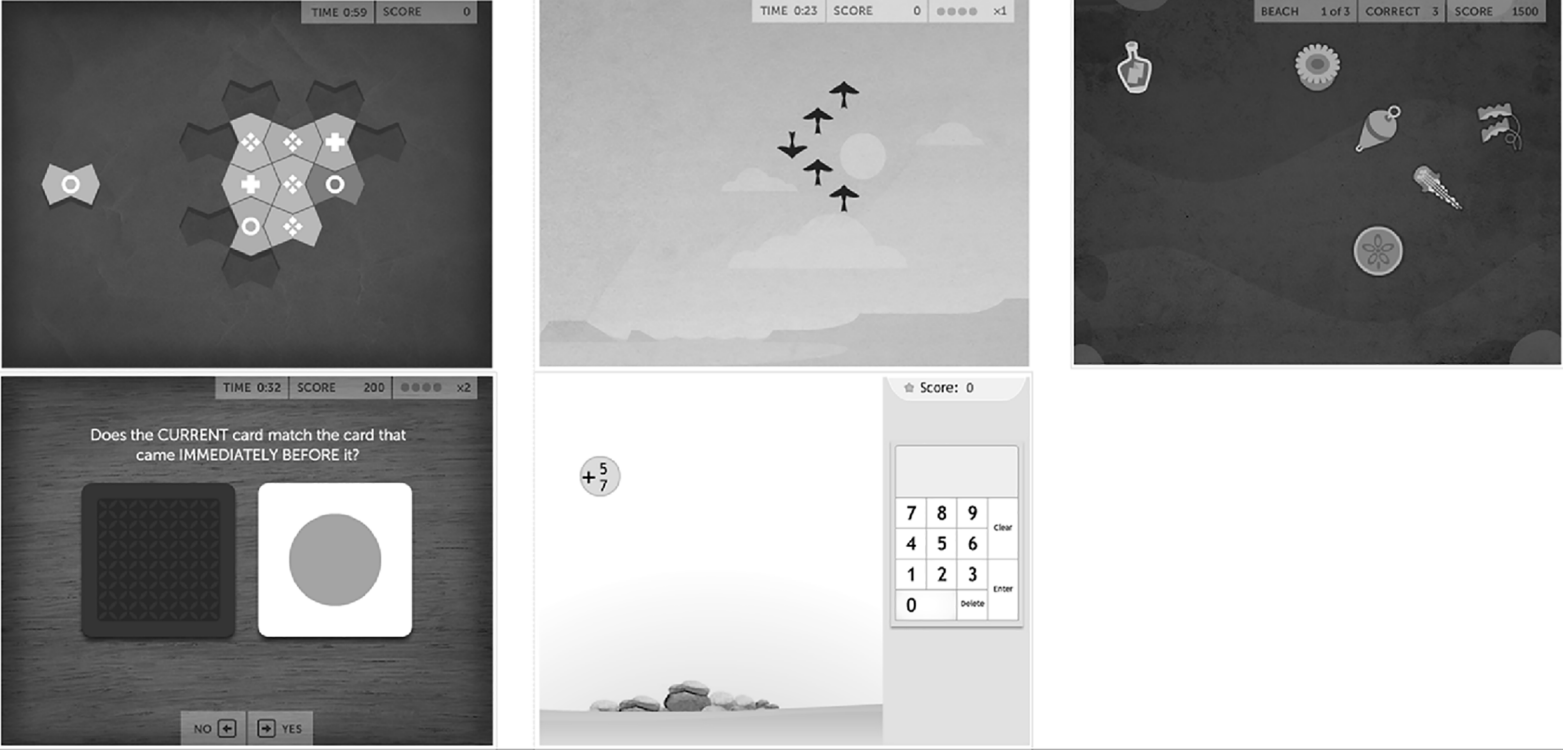
Screenshots of Lumosity Cognitive Training^TM^ games (http://www.lumosity.com). Legend. Top to bottom, left to right: Disillusion, Lost in Migration, Tidal Treasures, Speed Match, Raindrops.

For this study, 5 games were selected out of those available from the CCT, each stimulating a different cognitive domain. Playing the selected games did not require the mediation of language, as exercises proposed activities based on visual-spatial but not verbal information. Moreover, the games were considered by the research team to be easy to understand and perform. For an overview of the objectives, rules and screenshots of the games used in this study, see Table 1 and Fig 2.

**Table 1:** Games and objectives for each cognitive domain.

Name of games	Trained cognitive function(s)	Player goal/objective(s)
*Disillusion*	Cognitive flexibility	The child is asked to insert a tile in a matrix, matching it by symbol or color with another tile in light of the orientation of the target tile (horizontal or vertical). This exercise trains the ability to respond to a task modifying the rule of matching, based on contextual information. The more tiles the child is able to match the higher the score.
*TidalTreasure*	Visual-spatial memory	The child is presented with a beach where different objects appear. He/she has to select an object and then all objects are covered. In the subsequent screen he/she is asked to select an object that is different from the previous one and so on. Each session is composed of three beaches. The child fails when he/she selects a stimulus that was already chosen. The more objects the child selects the higher the score.
*Speed Match*	Processing speed and spatial working memory	The child has to indicate as quickly as possible whether a card is the same as the last one displayed, based on the symbol presented on it. As speed performance improves, the number of trials increases, increasing difficulty level. The more correct answers given, the higher the score.
*Lost in Migration*	Selective attention	The child is asked to indicate with the correct arrow key the direction of the central bird among a bird flock. Other birds are presented with the same or different direction from the central bird. The more correct answers given, the higher the score.
*Raindrops*	Arithmetic calculation	The child is asked to solve mathematical operations contained in rain-drops. He/she is required to give an answer before the raindrop falls into the sea at the bottom of the screen. The child is presented with three game possibilities within each session. The more correct calculations performed, the higher the score.

### Measures

Feasibility outcome measures (Table 2) were taken from a previous study [23] on a CCT for adolescents with brain damage, in order to replicate the same outcome measures and use a-priory defined criteria. Such criteria were based on the relevant literature on pilot feasibility studies [37–38].

**Table 2:** Feasibility outcome measures, taken and adapted from Verhelst et al. (2017) [33].

	*Feasibility measures*	*Feasibility questions*	*Data collected*	*Feasibility criterion for success*	*Outcome*	*Success*
Feasibility of intervention	accessibility	Do participants understand all game objectives and rules?	Number of participants who asked for further instructions to understand games when at home.	100% of participants understand all games	No participant, after being instructed in vivo and receiving written instructions for games, required further explications on games.	Yes
	training compliance	Will participants play all training sessions during the 8-week training period?	Mean percentage of sessions completed during the 8-week training period.	80% of training intervention is completed after 8 weeks	Average completion of 94.2%. A patient dropped out after 15 sessions. For 29 out the 31 patients who concluded the 8-week training period the completion range was 90.00%-100.0%. The remaining 2 patients completed 67.5% and 77.5% of training respectively.	Yes
	technical smoothness	Will there be no technical issues with the training material?	Number of participants who encountered technical issues that could generate a training interruption of > 3 days consecutively, possibly influencing total training duration.	100% of participants will be able to perform their training without technical issues	3 of the participants encountered a technical issue as a result of programming error. This issue was automatically resolved by the program within an hour, ensuring that participants could continue their training without any noteworthy interruption.	Yes
	training motivation	Will the participants be motivated to perform the training intervention?	Scores at an acceptability questionnaire on the training program.	80% of participants have a neutral or positive score on the global score of the questionnaire	28 out of 31 participants (90.32) who completed the 8-week training period showed neutral to positive global mean scores	Yes
Feasibility of study design and procedures	Participation willingness	What is the participation rate?	Number of participants who agreed to partake the training intervention among those who were contacted.	75% of eligible participants agree to take part in the study	32 out of 41 eligible participants (78%) agreed to take part in the study	Yes
	Participation rates	Do all eligible participants who agree to partake actually perform the training intervention?	Number of participants who agreed to take part and who actually performed the training intervention and number of children who abandoned the 8-week training.	80% of participants who agree to take part actually participate in the study	31 out of 32 of participants (96.9%) who agreed to partake actually completed the take part intervention. Only 1 patient dropped out in the middle of the training program due to lack of interest.	Yes
	Loss to follow-up	Can all data be collected without any problems?	Number of participants for whom all pre-treatment and post-treatment measures were collected.	90% of the outcome measures are collected	90.3% of the outcome measures were collected. For 3 participants we could not administer mathematical tasks, as they were not able to respond to requests (such tasks were not administered at pre-treatment as well)	Yes
	Assessment time scale	Can follow-up data be collected within a week after the 8-week training period?	Number of patients whose follow-up data were collected within a week after the 8-week training period.	Time from the end of training to first follow-up data collection <7 days for all participants	Post-training measurements of all participants were collected within 1 week after training	Yes
	Assessment procedures	Was the loss to follow-up acceptable?	Number of patients who failed to complete outcome measures at follow-ups.	Less than 20% of participants fail to complete outcome measures on post-training assessments	100% of participants who finished the intervention completed post-training assessments	Yes

Among these 9 outcome measures, 4 were related to the training intervention (accessibility, training compliance, technical smoothness, and training motivation) and 5 to the study design and procedures (participation willingness, participation rates, loss to follow-up, assessment timescale, and assessment procedures). In accordance with the previous study [23], the global criterion for intervention success required a score of 9/9 satisfied outcome measures.

The only difference with respect to the referred study [23] was that we administered a custom-made questionnaire (Table 3) to assess training compliance rather than the Intrinsic Motivation Inventory, as this questionnaire was not available in Italian. Scores of our custom-made questionnaire ranged from 1 (strongly disagree) to 5 (strongly agree) on a Likert scale; item scores ≥ 3 were considered as neutral or positive, so that, for each patient, a global score ≥ 15 was considered as a neutral or positive evaluation of training acceptability.

**Table 3:** Items and scores of the self-report questionnaire assessing training compliance.

			N = 31	
		M	(SD)
**Item**			
1	I appreciated taking part in the training project	3.68	0.98
2	I believe that my friends would like to take part in the training	3.45	1.18
3	It was simple for me to perform the games at the beginning of the training	3.68	1.08
4	It was hard to perform the games continuously during the 8-week training period	3.58	0.99
5	The games were not too complex to be correctly performed	3.77	0.94
**Total score**		18.77	3.12

1 = Strongly disagree; 2 = Disagree; 3 = Neutral; 4 = Agree; 5 = Strongly Agree.

Finally, to collect preliminary data on training outcome, we adopted the Lumosity Performance Index (LPI), which represents the “weighted average of performance across tasks based on percentiles for a given age group” [43] (p. 7394). This index was automatically supplied by the training web-platform and assessed changes in performance on trained exercises with practice.

### Statistical analysis

Descriptive statistics were used to describe demographic and clinical variables, feasibility and training outcome measures. Preliminary evaluation of the improvement in the trained exercises was conducted by comparing with paired-sample t-test (one-tailed) the LPI of participants who completed the training program (N = 31) between the first day and last day of training. We correlated the differences in LPI between the first and last day of training with Full Scale Intellectual Quotient (FSIQ) and age at training, using the Pearson’s correlation analysis. With respect to gender, the LPI changes of male and female patients were compared with independent-sample t-test (two-tailed). All analyses were performed with the SPSS 22.0 software.

## Results

### Recruitment

At the beginning of the study, we proposed participation to 41 families of eligible patients. Among them, 32 (78.0%) agreed to participate (see Fig 2). Reasons for declining participation were: inability to respect research programmed timelines due to distance from the rehabilitation center (55.6%), no interest in participating (33.3%), and excessive commitments of children (11.1%).

Demographic characteristics of the 32 participants are shown in Table 4. Nineteen participants were males and the prevalent diagnoses were brain trauma and brain tumors. The FSIQ was obtained for each participant during baseline assessment through the administration of the Wechsler Intelligence Scales for Children Fourth Edition (WISC IV; Wechsler, 2012) [44]. The average FSIQ of the sample was 89.3 (DS = 22.9), thus at the low end of the normal range. The socio-economic status (SES) of participant families was calculated in accordance with Hollingshead’s classification [45]. It ranged from a minimum of 1 to a maximum of 9 points. The average SES of participant’s families was located in the middle range (M = 5.4; DS = 2.0).

**Table 4:** Demographic and clinical characteristics of participants.

	N = 32			
	Mean	(SD)	n	(%)
Sex (males)			19	(59.4%)
Mean age (years)	13.5	(1.6)		
*11–12*			9	(28.1%)
*13–14*			12	(37.5%)
*15–16*			11	(34.4%)
Diagnosis				
*brain trauma*			10	(31.2%)
*ischemic and hemorrhagiclesion*			7	(21.9%)
*brain tumor*			11	(34.4%)
*cerebellarmalformation*			4	(12.5%)
FSIQ score	89.3	(22.9)		
*superior (120–129)*			2	(6.3%)
*high average (110–119)*			5	(15.6%)
*average (90–109)*			12	(37.5%)
*Low average (80–89)*			5	(15.6%)
*borderline (70–79)*			2	(6.3%)
*Extremely low (69 and below)*			6	(18.8%)
Family SES	5.4	(2.0)		

FSIQ = Full Scale Intellectual Quotient; SES = socioeconomic status.

### Feasibility outcome

Feasibility outcomes are presented in Table 2. All 4 criteria regarding training intervention were met: (1) all participants (100.00%) understood game goals and rules without requiring further explanations; (2) overall, 94.20% (SD = 16.00; range: 67.50–100.00%) of training sessions were completed after 8 weeks. One patient with a brain tumor aged 12 years and presenting a Full Scale Intellectual Quotient of 88 dropped out after performing 15 sessions, due to a lack of interest in the training program. 29 out of the 31 participants who concluded the 8-week training intervention (93.55%) carried out at least 90.00% of the training program (range = 90.00–100.00%), while 2 patients completed less than 80% of sessions (67.50% and 77.50% respectively). (3) 3 out of 32 patients (9.38%) encountered a technical issue as a result of a programming error, but the bug was automatically resolved by software developers a few hours later, ensuring that participants could continue the training program without any significant interruption. Thus, no noteworthy technical issue was registered; (4) regarding training compliance, 28 out of the 31 participants who completed the training program (90.30%) showed neutral to positive mean scores in the acceptability questionnaire. Means and standard deviations of scores in this questionnaire are shown in Table 3.

All 5 criteria concerning feasibility of study design and procedures were met: (1) 78.00% of eligible participants (32 out of 41) agreed to take part in the study; (2) 96.90% of them actually performed and completed the training program. Only one patient (3.10%) dropped out due to lack of interest in carrying out the training program; (3) for 90.30% of the participants (28 out of 31) who completed the training program, all efficacy outcome measures were collected at baseline and follow-up assessments. For 3 participants (9.70%) we could not collect all defined outcome measures, as they were not able to respond to requests associated with mathematical tests; (4) for all participants who finished the training program (100.00%) post-training data were collected within a week; (5) for all participants who finished the training program (100.00%) outcome measures were collected at both follow-up assessments.

### Training outcome

The mean LPI of the participants who completed the training program showed a significant increase between the first day (M = 682.58; SD = 202.43) and the last day (M = 917.03; SD = 363.60) of training (*t*(30) = 5.06, *p* < 0.001; Cohen’s *d* = 0.80).

We found a marginally significant correlation between increase in LPI and FSIQ (*r* = 0.35, *p* = 0.053), suggesting that individuals with higher intellectual abilities saw greater improvement from the CCT. No significant correlation (*r* = -0.01; *p* = 0.956) was found between LPI increase and age at training. No significant difference between male and female adolescents (*t*(29) = 1.17; *p* = 0.250) was found.

## Discussion

This study examined the feasibility of a home-based CCT in a sample of Italian adolescents aged 11–16 with a diagnosis of congenital or acquired brain damage. The program used for this study (Lumosity) was only available in English. Therefore, the evaluation of the feasibility of the training also included the testing of measures adopted to make the web-platform comprehensible and accessible to non-English speaking participants. The feasibility of study design and procedures was also evaluated. To test both these feasibility aspects, a series of previously set and validated measures [23] based on relevant literature [37–38] was used.

With respect to the feasibility of the training program, 93.55% of participants completed at least 90% of the training program after 8 weeks and the mean percentage of sessions completed was 94.2%. This demonstrates that telerehabilitation can be a suitable opportunity for pediatric patients with brain damage. With respect to motivation, in the self-reported questionnaire on training acceptability, most participants indicated positive commitment to the training program and reported high levels of perceived usefulness of the program. Therefore, the training program was considered to be sustainable and relevant. We believe that the weekly contact provided to participants and families by a research team member was crucial to sustain motivation for the training program. Indeed, it guaranteed compliance monitoring at an age where adolescents with numerous commitments and possible behavioral concerns may be less motivated to remain engaged in demanding activities. This hypothesis is in accordance with previous research [46–47] which reported that the presence of a coach seems to be a motivating factor for individuals undergoing rehabilitation treatments.

Even though the training was proposed in English to Italian native speakers, we observed that understanding game objectives and rules did not constitute a limiting issue for patients, as no one required instructions other than the ones directly received by the operators and the written instructions. This finding is particularly important with regards to clinical practice, as it highlights that the use of simple arrangements to overcome language barriers can have considerable success and allows for the possibility of using remote English trainings in non English-speaking contexts.

No significant technical issue interfering with training attendance was reported, demonstrating that the delivering of a CCT may fit well with the clinical needs of intensive and continuative cognitive stimulation of patients. As we involved a sample of patients not selected based on their intellectual functioning, our data can be considered particularly representative for the population of pediatric brain damaged patients, which is inhomogeneous in terms of cognitive performance level. This suggests that the selected training exercises may be successfully proposed for a remote intervention to patients with low intellectual ability. In our sample, 8 patients showed borderline or extremely low intellectual functioning and they all succeeded in performing the training program.

With regards to the feasibility of the study design and procedures, we registered high participation willingness, since more than ¾ of the contacted families accepted to participate. This suggests that parents of pediatric brain damaged patients are highly motivated to introduce new forms of remote cognitive stimulation in the daily routine of their children. With respect to participation rates, only one patient dropped out of the study. Based on the patient’s report, the reasons for leaving the study were associated with disinterest and not to excessive program challenges. The high rate of adherence to training is even more encouraging if we consider that the training duration was longer than for other programs for this population (8 weeks vs. 4–6 weeks of other trainings) [28–29].

Moreover, except for the adolescent who refused to continue the training program, for the other participants no loss at follow-up was registered, and for all patients who concluded the training program it was possible to collect follow-up data respecting the programmed timelines. This confirms that participants were highly compliant and performed all the programmed research steps. Therefore, this study seems to provide a reliable method to evaluate the effects of a remote CCT.

Useful considerations for clinical practice can be drawn by considering what happened with the 9 out of 41 families that did not agree to participate. It is noteworthy that the most frequent reason given to decline participation (55.6%) was the need to reach the Institute to perform neuropsychological assessments before and after training within a given timeline. This suggests that a higher percentage of families may accept to take part in a home-based CCT if less stringent demands related to evaluation timing are given. In order to control for this aspect, future studies could also enroll children of those families who declare themselves unable to adhere to the research timelines, excluding them from follow-up evaluations. Verifying training completion rates of children of these families could allow the control of whether difficulties related to research timelines and distance are real issues or a way to mask disinterest towards telerehabilitation.

While testing the efficacy and transferability of the program is beyond the scope of the present study, the preliminary results on change in cognitive performance after the training program are promising. This supports the fact that remote CCT may be useful in stimulating cognitive functioning in pediatric patients with brain damage. In particular, our preliminary data suggest that patients with higher intellectual functioning may gain greater benefits from CCT, while no effects of age and gender were observed. This suggests that CCT can be used with male and female adolescents of different ages.

There are several limitations to this study. First, no control group performing another CCT was included. This did not allow for the consideration of variations in adherence and satisfaction associated with specific training characteristics. In a similar vein, we could not control for the role of the support of a research member in the form of weekly contact in facilitating the compliance with the study protocol. Future studies should compare the CCT with an active control program that includes similar involvement of a researcher providing encouragement and supervision, in order to test the acceptability aspects that are specific for the training program. Second, even though our sample included heterogeneous diagnoses and thus was representative of the wide population of individuals with brain damage, the variety of etiologies of brain damage and their inhomogeneous numerical distribution could have masked issues faced by a specific group of patients. Future studies could benefit from including a higher number of participants and examining the implications of different diagnoses on outcomes. In particular, in this study the group of patients with congenital brain damage was very limited (N = 4), making the results limitedly generalizable to this population. Moreover, feasibility was assessed in a group of adolescents of motivated families. It could be that the percentage of adherence is less when applied to the general population of pediatric patients with brain damage. This aspect should be verified in future research by evaluating adherence to a CCT proposed as routine clinical practice. Finally, even though in our study training duration was more prolonged as compared to the one reported by previous studies [28–29], we were not able to provide information on the response of patients at more extended time points. Finally, the training program was a novelty for enrolled adolescents and it is possible that the percentage of adherence in response to the repetition of the program could be lower over time. Subsequent studies should monitor compliance over time and provide suggestions on how to maintain compliance.

### Conclusions

In conclusion, this study demonstrated the feasibility of a home-based CCT in adolescents with congenital or acquired brain damage and various levels of cognitive functioning. Thus, such an intervention proposal may represent an accessible opportunity for rehabilitation among this population. The CCT was not in the participants’ mother tongue, but the language barrier was successfully overcome through simple arrangements. This finding provided important indications on the possibility of introducing telerehabilitation interventions in those countries where few or no home-based cognitive programs in mother tongues are available. Given these considerations, we recommend the continuation of studies on telerehabilitation protocols applied to brain damaged patients. This study involved a mixed sample of patients in relation to etiology. For future studies, it could be worthwhile to examine the effects of this variable on training adherence. This will provide evidence-based support to this potentially important new path for neurorehabilitation. The research protocol used for this study can be a viable method to conduct investigations on this issue.

## Supporting information

S1 Protocol Home-based computerized cognitive training for pediatric patients with congenital or acquired brain injury: A randomized controlled trial. (DOCX)

S1 Checklist CONSORT 2010 checklist. (DOC)
