# Supplementary material for: Feasibility of a home-based computerized cognitive training for pediatric patients with congenital or acquired brain damage: An explorative study
Source: PLoS One. 2018 Jun 20;13(6):e0199001. doi: 10.1371/journal.pone.0199001 (PMC6010294; doi:10.1371/journal.pone.0199001)
Supplement: S1 Protocol — (DOCX) [file pone.0199001.s001.docx]

**S1 Protocol. Home-based computerized cognitive training for pediatric patients with congenital or acquired brain injury: a randomized controlled trial**

**Introduction**

A congenital or acquired brain injury may have a dramatic impact on children’s quality of life and development. Cognitive deficits are one of the most disabling long-term consequences of such a disease, due to damage to the central nervous system in a maturational state (Malia et al., 2004; Tavano et al., 2007; Mulhern & Palmer, 2003). Anomalies in neural plasticity and progressive modularization and specialization of cerebral networks may generate deficits in global intelligence or single domains such as attention, memory, language, executive functions, problem-solving and visual-motor competences ([Anderson, Catroppa, Dudgeon, Morse, Haritou, & Rosenfeld, 2006](http://www.ncbi.nlm.nih.gov/pmc/articles/PMC2733858/#R3); [Ewing-Cobbs, Barnes, Fletcher, Levin, Swank & Song, 2004](http://www.ncbi.nlm.nih.gov/pmc/articles/PMC2733858/#R42);  [Fay, Jaffe, Polissar, Liao, Rivara, & Martin, 1994](http://www.ncbi.nlm.nih.gov/pmc/articles/PMC2733858/#R46); [Levin, Ewing-Cobbs, & Eisenberg, 1995](http://www.ncbi.nlm.nih.gov/pmc/articles/PMC2733858/#R69); Mulhern & Palmer, 2003; [Taylor, Yeates, Wade, Drotar, Stancin & Minich, 2002](http://www.ncbi.nlm.nih.gov/pmc/articles/PMC2733858/#R94); [Yeates, 2000](http://www.ncbi.nlm.nih.gov/pmc/articles/PMC2733858/#R104); Tavano et al., 2007). These difficulties may have multiple cascade effects, impacting on school performance, social functioning and quality of life of patients and generating problematic adjustment (Arroyos-Jurado, Paulsen, Merrell, & Lindgren, 2000; Ewing-Cobbs et al., 2004; Souza, Braga, Filho, & Dellatolas, 2007).

In front of the high occurrence of cognitive impairment and associated lifetime costs in children with brain injury, cognitive rehabilitation has been established to be a very need in order to minimize ongoing problems and limit long-term cognitive decay (Anderson & Catroppa, 2006; Bardoni, Galbiati, Recla, Pastore, Formica & Strazzer, 2013; Cicerone et al., 2000). Numerous studies documented this kind of treatment being effective in improving patients’ functioning (e.g., Butler et al., 2008; Cicerone et al., 2000; Laatsch et al., 2007). As reported by Cicerone and colleagues (2000), “cognitive rehabilitation services should be directed at achieving changes that improve each person’s function in areas that are relevant to their everyday lives” (p. 1597). It was documented that the efficacy of rehabilitation treatments increases if programs start as soon as possible, are intensive as much as possible and are prolonged during the recovery phase at home (Zampolini et al., 2008). These findings indicate there is the need to guarantee early tailored rehabilitation opportunities to patients. Conversely, in absence of rehabilitation, patients may not achieve their optimal level of functioning or may be at greater risk of cognitive decay over time.

Traditional cognitive rehabilitation is performed at specialized centers, where face-to-face or group interventions are delivered (Kueider, Parisi, Gross, & Rebok, 2012). However this kind of intervention presents with some limitations, since it tends to be time-limited, costly, impractical to the majority of patients and does not guarantee treatment homogeneity across clinicians (Cicerone et al., 2000; Di Scala, Osberg, & Savage, 1997; How, Hwang, Green & Mihailidis, 2016; Kesler Lacayo, & Jo, 2011; Schmeler, Schein, McCue, & Betz, 2009). Recently, new rehabilitation programs based on technological devices to be used at home or out of the medical setting have been introduced in the clinical practice in order to increase opportunities and standards of rehabilitation. These new practices are defined as telerehabilitation, namely the application of telecommunication technology to provide at distance care to individuals with disabilities (Ricker et al., 2002; Schmeler et al., 2009).

Telerehabilitation offers different benefits: 1) it enables individuals with disabilities to gain access to rehabilitation services, regardless of the limitations imposed by geography and local resource capabilities (Schmeler et al., 2009); 2) it guarantees interventions to patients after discharge from hospital, enabling continuity of care (Zampolini et al., 2008); 3) it consents remote contact between clinicians and patients, which is associated to diminished access to hospitals (Zampolini et al., 2008); 4) it empowers patients to take control of the management of their medical needs and interventions, reducing economical demands and improving motivation and self-agency (Brennan, Mawson, & Brownsell, 2009) 5) it allows clinicians to monitor at distance exercises execution and obtain automatically performance parameters, with reduction of costs and resources required to sustain rehabilitative interventions (Alloni et al., 2015; Zampolini et al., 2008); 6) due to the accurate tracking of performance parameters, it allows quantifying the benefits of treatments and evaluate their real efficacy, as requested (Robertson & Fitzpatrick, 2008; Slomine & Locascio, 2009). Therefore, the introduction of telerehabilitation may have a positive impact on patients, healthcare systems and scientific community.

Studies on the accessibility and efficacy of telerehabilitation programs to stimulate/develop cognitive functions in pediatric patients with a brain injury are still limited (Brennan et al. 2009; Tam et al., 2003), but results are promising (Conklin et al., 2015; Kesler et al., 2011; Kurowski, Wade, Kirkwood, Brown, Stancin, & Taylor, 2013; Kurowski, Wade, Kirkwood, Brown, Stancin, & Taylor, 2014; Madsen Sjö, Spellerberg, Weidner, & Kihlgren, 2009; van't Hooft, Andersson, Sejersen, Bartfai, & von Wendt, 2003; van‘t Hooft, Andersson, Bergman, Sejersen, von Wendt, & Bartfai, 2007; Wade, Stancin, Kirkwood, Brown, McMullen, & Taylor, 2014; Wade, Walz, Carey, & Williams, 2008). Given such considerations, the present study aims at further investigating the accessibility and efficacy of a home-based cognitive training in a group of brain injured children. This goal is important to increase knowledge on potential remediation alternatives for this clinical population to be inserted in the clinical practice.

A distinctive aspect of this research is that it aims at examining this issue in a population of non-English speaking patients presented with a web-platform in English, while previous research has involved children whose mother tongue was English or who were proposed a training in their mother tongue (e.g., Hendricks, 1996; van’t Hooft et al., 2003; 2007). The importance of such an investigation is related to two considerations. First, as most on-line cognitive programs are provided in English, language may represent an obstacle to implementation of telerehabilitation in non-English speaking countries, making these nations to have fewer possibilities to guarantee a continuative and well-advanced care to patients. For this reason, it is paramount to comprehend if language may effectively be a limitation to telerehabilitation delivery or if the usage of simple strategies, such as the selection of specific non language-mediated exercises and the provision of instructions in native language, may help to overcome this impasse. Second, in countries where telerehabilitation is not yet diffuse and well-established, this intervention may encounter more resistance by children and families as compared to countries where it is commonly provided. Thus, in order to understand the potentialities of this form of rehabilitation in countries where it is a new opportunity, it is essential to evaluate the percentages of families that accept its offer, the reasons of refusals and the degree of adherence to training.

To these aims, we proposed a randomized clinical trial to get controlled data on both feasibility and efficacy, in consideration of the need to provide higher quality research in the rehabilitation field (Robertson & Fitzpatrick, 2008) and develop well-designed studies to examine the efficacy of interventions in the population of children with brain damage (Slomine & Locascio, 2009).

**Research objectives and hypotheses**

We aim to investigate the feasibility and efficacy of a 40-session Lumosity cognitive training in a sample of Italian patients aged 11-16 with acquired or congenital brain injury.

As regards to efficacy, we want to estimate both neurocognitive and functional adjustment outcomes, since previous research reported that in this population neuropsychological functioning is strictly related to global functioning (Arroyos-Jurado et al., 2000; Ewing-Cobbs et al., 2004; Souza et al., 2007).

We hypothesize that Lumosity cognitive training may:

- result feasible to a population of pediatric non-English speaking brain injured patients, after a precise selection of the exercises by the research team and provision of instructions in original mother tongue
- produce benefits in ameliorating cognitive performance
- generate improvement of patients’ quality of life and adjustment

**Methods/Design**

As the research involves underage children, subjects are included in the project after written informed consent is obtained from their parents.

Design

This study is a single-center clinical controlled trial applying a stepped-wedge research design. This design allows comparing two groups of patients assigned to two different conditions: the first group (G1) receives the non-treatment first, followed by the cognitive training, while the other group (G2) receives the training first, followed by the non-treatment. This design allows taking into account spontaneous cognitive maturation and education effects, granting internal validity to the study and, at the same time, it addresses ethical issues about the need to grant all patients the same rehabilitation opportunities. Such a design has already been proposed in a previous randomized controlled trial on the effectiveness of a computerized cognitive training in children with neurodevelopmental disorders (Løhaugen et al., 2014.), thus it seems to fit well with the aims of the present study.

Practically, all enrolled participants are initially evaluated through a battery of neurocognitive tests and questionnaires on adjustment (T1). Then, participants are randomized into 2 groups. Children of G1 immediately start the 2 month-training (step 1) and are re-evaluated at T2, in order to assess the effects of the treatment. Then, they enter a non-treatment period of 2 months (step 2). For G2 the 2 steps are inverted: at step 1 children wait and serve as control, while at step 2 they start the training. At T3, G2 are evaluated after treatment, while G1 after an equivalent period of non-treatment after the end of the cognitive training. After 6 months from the end of the treatment, a follow-up assessment is performed for both G1 (T4) and G2 (T5), in order to check for long-lasting effects of treatment. A flowchart of the study is reported in Figure 1.

Participants and testers are blinded to allocation to groups: the research is a double-blind trial. This trial is conducted and reported in accordance to CONSORT guidelines for non-pharmacological interventions (Boutron, Moher, Altman, Schulz & Ravaud, 2008; Campbell, Elbourne, & Altman, 2004).

Fig A. Flowchart of the study.


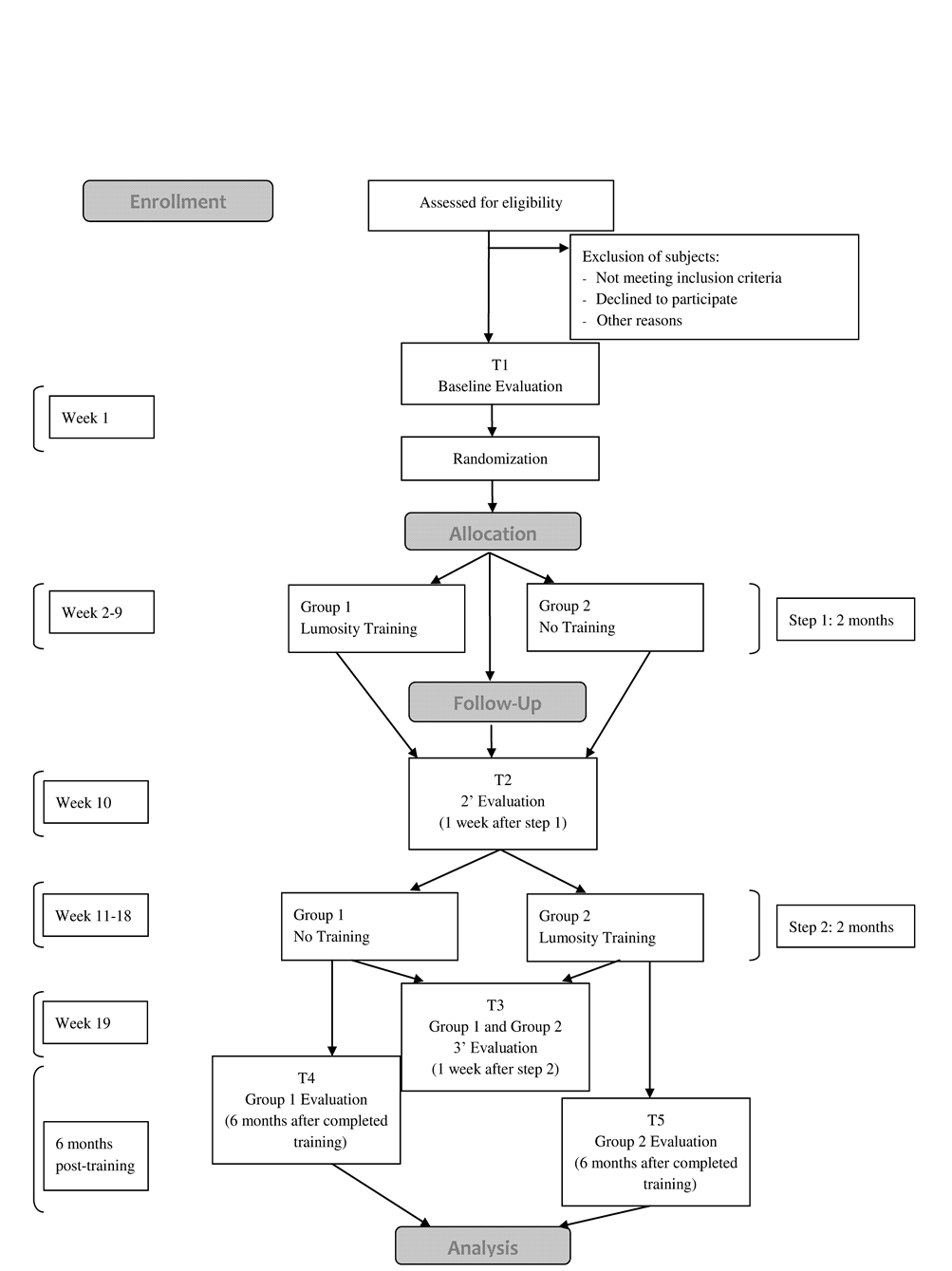


Participant sample and recruitment and eligibility criteria

Participants are recruited at the Scientific Institute IRCCS E. Medea, among children with a congenital or acquired brain injury in chronic phase who are referred to the Neurorehabilitation Units of the Institute. Inclusion criteria are: an age between 11 and 16 years, as this age range represents an interval when children are all attending secondary school (of first or second level) according to the Italian academic system and cognitive requests and daily activities are usually elevated, so that traditional rehabilitation may cause high organizational costs to both the adolescent and his family; speaking Italian as a mother tongue, as instructions on the training to both children and families are provided in Italian. Exclusion criteria are: a previous psychiatric or cognitive diagnosis for children with an acquired brain injury, as it would not ensure homogeneity to the study sample; severe visual, auditory or motor deficits that could interfere with training execution and assessment; a parallel cognitive rehabilitation treatment, to avoid confounding effects of two interventions delivered in the same time-frame; a diagnosis of photosensitive epilepsy, as a computer-based stimulation may produce negative health effects.

No selection based on cognitive performance at baseline is made, as the study is intended to investigate the effects of an on-line training in the general population of children with brain injury in chronic phase. Level of functioning is considered for data analysis as a variable possibly influencing the effects.

Recruitment

Patients fulfilling research inclusion criteria are identified from the patients’ database of the Neurorehabilitation Units of our Institute. The referring clinician contacts the parents of eligible patients to propose the research project and give advance notice of the contact by a member of the research team. Then a member of the research team contacts the parents explaining the project objectives and methodology and informing them that, in case of assent, they are requested to complete and send back within a month the informed consent form. Children whose parents send back the document are considered enrolled in the project.

Overall trial start date: 01/01/2016. Recruitment start date: 02/03/2016 for the study on feasibility, after the approval of the project by the Ethics Committee of Scientific Institute IRCCS E. Medea, Bosisio Parini, Italy (project number 284) and 18/11/2016 for the study on efficacy, after the approval of the project as clinical trial by the Italian Ministry of Health (protocol number 44249 of 08/09/2016, approved on 18/11/2016).

Randomization and blinding

All children who have a completed informed consent are individually randomized into G1 or G2. The randomization is conducted by a researcher of the Institute, independently from the research team responsible for testing participants. Randomization of patient assignment followed a coin flip procedure using the randomization tool of Microsoft Excel; a random number is randomly associated to any recruited patient and determines assignment to G1 (0 to 0.49) or G2 (0.50 to 1). After randomization, parents are contacted by a researcher team member and receive information on the study steps. Participants and testers are blinded to allocation to groups.

Intervention

As intervention, we selected Lumosity Cognitive Training (Lumos Labs, 2010) in consideration of the following motivations: i) it allows the stimulation of different cognitive domains, which are frequently compromised in patients with brain injury; ii) it is adaptive, modifying the difficulty level of exercises on the individual performance, fact that is a need when the cognitive level of participants is inhomogeneous, thus possibly limiting drop-outs and adverse effects due to frustration; iii) it consents an intensive daily training but of a limited duration, sparing patients from excessive cognitive requests at an age where context demands (school and leisure activities) are elevated; iv) it allows the monitoring of patients performance through quantitative data provided at the end of each training session; v) it has been already studied in different clinical populations with positive conclusions, fact that is requested when a training is proposed to a clinical population receiving the parallel request to avoid any other form of rehabilitation during study duration.

Participants are provided free access to the Lumosity software. They access the cognitive platform through a personal username and a password, provided by the research team. Before starting the training, all participants receive direct demonstration of how to carry out the exercises and are given a written instructions on the exercises in Italian language. During the intervention, participants are asked to complete 40-sessions at home: they are expected to undergo a 20-minute training once per working day for 8 weeks. A weekly telephone-based contact with parents is provided, aimed at motivating them in sustaining children’s training adherence and at recording the reason of any drop-outs. The abilities targeted by cognitive training are: cognitive flexibility, memory, speed, attention and problem-solving.

The following games of Lumosity cognitive training have been chosen for the intervention:

• Disillusion (cognitive flexibility): the child is requested to insert a form in a matrix, matching it by symbol or color to another form, in view of the orientation of the form (horizontal or vertical). This exercise trains the ability to respond to a task modifying the rule of matching on the basis of contextual information. The more forms the child is able to match the higher the score.

• Tidal Treasure (visual-spatial memory): the child is presented with a beach where different objects appear. He/she has to select an object and then all objects are covered. In the subsequent screen he/she is requested to select an object that is different from the previous one and so on. Each session is composed of three beaches. Children fails when he selects a stimulus already chosen. The more objects the child selects the higher the score.

• Speed Match (processing speed and spatial working memory): the child has to indicate if a stimulus matches the previous one, in terms of the symbol presented on. As the speed performance improves, the number of trials increase, augmenting difficulty level. The more correct answers are given, the higher the score.

• Lost in Migration (selective attention): among a birds’ flock, the child is requested to indicate with the arrow keys the direction of the central bird. Other birds are presented with a same or a different direction from the central bird. The more correct answers he/she gives, the higher the score.

• Raindrops (arithmetic calculation): the child is requested to solve mathematical operations contained in rain-drops. He/she is asked to give an answer before that the raindrop falls into the sea at the bottom of the screen. He/she is presented with three game possibilities in each session. The more correct calculations the child does, the higher the score.

Measures

For all participants, baseline intellectual indices are assessed at T1 to verify the influence of the intellectual level on changes generated by the training. The Italian version of the Wechsler Intelligence Scales for Children IV Edition (WISC IV; Wechsler, 2012) is used to assess intellectual functioning.

In order to assess training adherence we collect:

• Number of dropouts: number of children who renounce to complete the 8-week training

• Number of sessions completed per child: total number of sessions performed in front of the expected total number of 40 sessions.

In order to assess training acceptability we administer an ad-hoc 5 item-questionnaire.

Primary outcome measure:

As primary outcome measure we consider visual-spatial working memory, as assessed by Corsi block tapping task (Corsi, 1972). Indeed, all selected exercises of the training require the manipulation of the visual-spatial information and all of them, except for the game “Lost in Migration” stimulating attention. At the Corsi block tapping task (Corsi, 1972), children are asked to indicate a visual-spatial sequence in the same order it was presented by an examinator. A z score will be collected for performance.

Secondary outcome measures:

As secondary outcome measures we consider both cognitive and adjustment measures .

Cognitive Measures:

• Cognitive flexibility: the Wisconsin Card Sorting Test (WCST; Grant & Berg, 1993) is administered. In this test, children are asked to identify a rule for associating cards and then to modify it on the basis of a computerized feedback. WCST measures difficulties in selecting flexible strategies and in blocking an automatic responding, abilities that are central for flexible thinking. Adjusted standard scores for number of total errors, perseverative responsive and perseverative errors are collected.

• Attention: the visual attention task of Conners’ Continuous Performance Test III (Conners, 2014) is performed. This attentional task asks children to press a button on the computer keyboard response to the presentation of any alphabetical letters comparing on the screen, except letter X. Omissions, commissions and perseverations at this task are automatically counted by the program. Adjusted T-scores for these measures are considered for this study.

• Speed: the reaction time index (HRT) of Conners’ Continuous Performance Test III (Conners, 2014), measuring response time during the visual attention task described above, is used. Adjusted T scores are collected.

• Problem-solving: an age-appropriate problem-solving task and an arithmetic calculation task of the Italian battery AC-MT (Cornoldi & Cazzola, 2003; Cornoldi, Lucangeli, & Bellina, 2002; Cornoldi, Pra Baldi, & Friso, 2010) for testing mathematical abilities is used. Z scores are collected.

Adjustment:

• Psychological adjustment: Child Behavior Checklist 6-18 (Achenbach & Rescorla, 2001; http://www.aseba.org) completed by parents, Youth Self-report 11-18 (Achenbach, 1991; http://www.aseba.org) completed by children and Teacher Report Form (Achenbach & Rescorla, 2001; http://www.aseba.org ) completed by teachers are used. These instruments yield scores on internalizing, externalizing and total psychological problems of children.

• Overall functioning and quality of life: The World Health Organization Quality of Life- Brief Version (WHOQOL; The WHOQOL Group, 1994) assessing quality of life, health and well-being of patients, is administered to children.

• Self-esteem: We administer the Italian version of the “Multidimensional Self-Concept Scale” (Bracken, 1992), which assesses self-concept related to the following six domains: social, competence, affect, academic, family and physical.

Outcome measures are collected in all assessment points for both G1 (T1; T2; T3; T4) and G2 (T1; T2; T3; T5).

Sample Size Rationale

A final sample of 60 patients is set for such a study in order to detect within-group change of moderate effect size (Cohen’s d = 0.47) (see Bowen et al., 2009) with a power of 0.95 and alfa level set at p < 0.05. The software G Power 3 (Faul, Erdfelder, Lang, & Buchner, 2007) was used for this estimation.

**Statistical Analysis**

The global study sample and each study group will be described with respect to demographic variables such as age, gender and socioeconomic status of families (SES) with descriptive statistics.

T-test and χ² analyses will be conducted to compare quantitative and qualitative variables, respectively, between the two groups of patients (i.e., G1 and G2).

Repeated-measures linear general model statistics will be used to compare cognitive measures between the two different time points, while controlling for individual intellectual functioning.

All analyses will be performed with the SPSS 22.0 software.

**Discussion**

Cognitive rehabilitation is highly recommended for pediatric patients with a brain injury to limit the progressive deterioration of performance over time and to reduce the associated vocational, social and psychological costs (Chevignard, 2016; Mostow, Walker, Frappaz, & Mulvihill, 1999; Tavano, 2007). Telerehabilitation represents a new form of rehabilitative service that may overcome problems associated to face to-face rehabilitation, such as limitation of taking in charge, elevated costs for families and hospitals, lack of control on interventions’ tailoring and efficacy, grating more and better care opportunities to patients.

For the specific population of pediatric children with brain injury, the most common telerehabilitation interventions for cognition consisted of computerized programs aimed at improving specific core cognitive abilities These programs enter Level B of treatments for children with brain injury, according to Limond’s model for neurocognitive intervention: this level is aimed at a remediation of skills (Limond, Adlam, & Cormack, 2014). As argued by authors, rehabilitation at this level is necessary to maximize basic skills before providing interventions on higher-level abilities since it may grant to those complex interventions higher long-term benefits. Studies on telerehabilitation programs demonstrated evidence of their accessibility and efficacy (Kueider et al., 2012), supporting their usage in combination and addition to traditional rehabilitation forms, in order to ensure a better quality of life of patients. However, further research is required on this issue, since studies on telerehabilitation at developmental age are currently few and with a bare control (dos Santos et al., 2014).

The present study aims at extending previous research through the investigation of the feasibility and efficacy of Lumosity cognitive training in a sample of Italian pediatric patients aged 11-16 years. Such an intervention is aimed at a remediation of skills. We propose a randomized controlled trial in order to guarantee an adequate control on data and findings, in respect with recommendations proposed for research on rehabilitation interventions (Teasell et al., 2007). Data of this randomized controlled trial (RCT) will allow researchers and clinicians to get worthwhile indications on directions to take about investments in the field of telerehabilitation. Indeed, if the training proposed by the present study demonstrates to be effective, useful data on a possible evidenced based intervention to be inserted in the ordinary rehabilitation course will be provided.

As this research proposes a training from a web platform written in English to Italian-speaking children, findings from this study may provide significant data on the usage of a web-based training in those countries where telerehabilitation is limited and few programs are available in the native language. The usage of simple arrangements, such as the selection of non-language-mediated exercises and the provision of instructions in the native language, is tested. This may allow us to get indications on the possibility to override obstacles to the implementation of telerehabilitation in countries where it still encounters considerable barriers and resistance due to its novelty.

Another peculiar characteristic of the present study is that it proposes the evaluation of the efficacy of a web-training stimulating various core cognitive domains, differently from most of previous trainings aimed at exerting only a specific cognitive function. The evidence about both the interdependence of different cognitive systems (Spevack, 2007; Goswami, 2008, Johnson, Hailt, Grice, & Karmiloff-Smith, 2002) and the frequent observation of diffuse cognitive effects of a brain injury, led us to consider essential to investigate the effects of a multiple-domain stimulation. Indeed, such a stimulation may be the most likely mean for having the greatest impact on cognitive outcome in these patients. For this reason, data of this research may provide an important contribution on interventions’ structuring. In fact, in case of favorable outcomes, rehabilitators may consider to structure programs where different core skills are contemporarily targeted by a unique training, with evident implications on time and economic resources allocation.

Globally, such a study will provide important knowledge on the feasibility and efficacy of a web-based cognitive intervention directed to children with a brain injury through a RCT. This will contribute to establish empirically-based recommendations for the practice of cognitive rehabilitation in such a population. Moreover, important indications on the possibility to introduce telerehabilitation as a care practice in non-English speaking contexts will be provided. Finally, effects on cognition of a program offering multiple-domain stimulation will be collected, providing significant methodological issues on interventions’ structuring. These data are paramount to establish appropriate planning and allocation of resources in the telerehabilitation field, in front of the very need of brain injured patients to be sustained in their cognitive functioning to reach an acceptable quality of life.

**Funders**

Our work is supported by the Italian Ministry of Health (Ricerca Corrente grants 2015-2017 to Alessandra Bardoni and Ricerca Finalizzata grant NET-2013-02356160 to Renato Borgatti). The funders will have no role in study design, data collection and analysis, decision to publish, or preparation of the manuscript.

**References**

1. Achenbach, T. M. (1991). *Integrative guide for the 1991 CBCL/4-18,YSR and TRF profiles.* Burlington: Department of Psychiatry, University of Vermont.
2. Achenbach, T. M., Rescorla, L. A. (2001). *Manual for the ASEBA school-age forms & profiles*. Burlington, VT: University of Vermont, Research Center for Children, Youth, & families.
3. Alloni, A., Sinforiani, E., Zucchella, C., Sandrini, G., Bernini, S., Cattani, B., … Pistarini, C. (2015). Computer-based cognitive rehabilitation: the CoRe system. *Disability and Rehabilitaion, 27*, 1-11.
4. Anderson, V., & Catroppa, C. (2006). Advances in post-acute rehabilitation after childhood-acquired brain injury: A focus on cognitive, behavioral and social domains. *American Journal of Physical Medicine and Rehabilitation, 85*, 767-778.
5. Anderson, V.A., Catroppa, C., Dudgeon, P., Morse, S.A., Haritou, F., & Rosenfeld, J.V. (2006). Understanding predictors of functional recovery and outcome 30 months following early childhood head injury. *Neuropsychology, 20*, 42–57.
6. Arroyos-Jurado, E., Paulsen, J. S., Merrell, K. W., & Lindgren, S. D. (2000). Traumatic Brain Injury in School-Age Children. Academic and Social Outcome. *Journal of School Psychology, 38*(6), 571–587.
7. Bardoni, A., Galbiati, S., Recla, M., Pastore, V., Formica, F., & Strazzer, S. (2013). Evolution of the cognitive profile in school-aged patients with severe TBI during the first 2 years of neurorehabilitation. *Brain Injury 27(12)*, 1395-1401.
8. Boutron, I., Moher, D., Altman, D. G., Schulz., K. F., & Ravaud, P. (2008). Extending the CONSORT statement to randomized trials of nonpharmacologic treatment: explanation and elaboration. *Annals of Internal Medicine, 148(4)*: 295.
9. Bowen, D. J., Kreuter, M., Spring, B., Cofta-Woerpel, L., Linnan, L., Weiner, D., ... Fernandez, M. (2009). How We Design Feasibility Studies. *American Journal of Preventive Medicine, 36*, 452–57.
10. Bracken, B. A. (1992). Multidimensional Self-Concept Scale. Austin, TX: Pro-Ed.
11. Brennan, D., Mawson, S., & Brownsell, S. (2009). Telerehabilitation: enabling the remote delivery of healthcare, rehabilitation and self management. In A. Gaggioli (Ed.), *Advanced Technologies in Rehabilitation* (pp. 231-248). Amsterdam: IOS Press.
12. Butler, R. W., Copeland, D. R., Furlough, D. L., Mulhern, R. K., Katz, E. R., Kazak, A. E., … Sahler, O. J. (2008). A multicenter randomized clinical trial of a cognitive remediation program for childhood survivors of a pediatric malignancy. *Journal of Consulting and Clinical Psychology, 76*, 367-378.
13. Campbell, M., Elbourne, D., & Altman, D. (2004). CONSORT statement: extension to cluster randomised trials. *British Medical Journal, 328(7441)*: 702.
14. Chevignard, M. (2016). Children with brain tumours need long-term multidisciplinary psychosocial, neurocognitive, academic and rehabilitation follow-up programmes. *Acta Paediatrica, 105(6)*, 574-575.
15. Cicerone, K. D., Dahlberg, C., Kalmar, K., Langenbahn, D. M., Malec, J. F., Bergquist, T. F., … Morse, P. A. (2000). Evidence based cognitive rehabilitation: recommendations for clinical practice. *Archives of Physical Medicine and Rehabilitation, 81*, 1596-1615.
16. Conklin, H. M., Ogg, R. J., Ashford, J. M., Scoggins, M. A., Zou, P., Kellie, N., … Zhang, H. (2015). Computerized Cognitive Training for Amelioration of Cognitive Late Effects Among Childhood Cancer Survivors: A Randomized Controlled Trial. *Journal of Clinical Oncology, 33*, 3894-3902.
17. Conners, C. K. (2014). *Conners Continuous Performance Test- Third Edition (Conners CPT 3) & Conners Continuous Auditory Test of Attention (Conners CATA): Technical Manual*. New York: Multi-Health Systems Inc.
18. Cornoldi, C., & Cazzola, C. (2003). AC-MT 11-14. *Test di valutazione delle abilità di calcolo e problem-solving dagli 11 ai 14 anni*. [AC-MT 11-14. Test for evaluating arithmetic and problem-solving abilities]. Trento: Erickson.
19. Cornoldi, C., Lucangeli, D., & Bellina, M. (2002). *Test AC-MT 6-11 - Test di valutazione delle abilità di calcolo.* [AC-MT 6-11 test – Test for evaluating calculation abilities]. Gruppo MT. Trento: Erickson.
20. Cornoldi, C., Pra Baldi, A., & Friso, G. (2010). *MT Avanzate – 2. Prove MT Avanzate di Lettura e Matematica 2 per il biennio della scuola superiore di II grado.* [Advanced MT – 2. Advanced MT reading and mathematical tasks for the second grade of secondary school]. Firenze: Organizzazioni Speciali.
21. Corsi, P.M. (1972). *Human memory and the medial temporal region of the brain*. Thesis (PhD). McGill University. Available at: http://digitool.library.mcgill.ca/webclient/StreamGate?folder_id=0&dvs=1507640614378~22
22. Di Scala, C., Osberg, S., & Savage, R.C. (1997). Children hospitalized for traumatic brain injury: Transition to post-acute care. *Journal of Head Trauma and Rehabilitation, 12*, 1-10.
23. dos Santos, M. T. N., Moura, S. C., Gomes, L. M., Lima, A. H., Moreira, R. s, Silva, C. D., Guimarães, E. M. (2014). Telehealth application on the rehabilitation of children and adolescents. *Revista Paul Pediatrica, 32(1)*, 136-143.
24. Ewing-Cobbs, L., Barnes, M., Fletcher, J. M., Levin, H. S., Swank, P. R., & Song, J. (2004). Modeling of longitudinal academic achievement scores after pediatric traumatic brain injury. *Developmental Neuropsychology, 25*(1&2), 107–133.
25. Ewing-Cobbs, L., Prasad, M.R., Landry, S.H., Kramer, L., & DeLeon, R. (2004). Executive functions following traumatic brain injury in young children: A preliminary analysis. *Developmental Neuropsychology, 26*, 487–512.
26. Faul, F., Erdfelder, E., Lang, A. G., Buchner, A. G. (2007). Power 3: A flexible statistical power analysis program for the social, behavioral, and biomedical sciences. *Behavioral Research and Methods, 39*, 175-91.
27. Fay, G.C., Jaffe, K.M., Polissar, M.L., Liao, S., Rivara, J.B., & Martin, K.M. (1994). Outcome of pediatric traumatic brain injury at three years: A cohort study. *Archives of Physical Medicine and Rehabilitation, 75*, 733–741.
28. Goswami, U. (2008). *Cognitive development: The learning brain*. Hove, UK: Psychological Press.
29. Grant, D. A., & Berg, E. A. (1993). *Wisconsin Card Sorting Test*. Odessa (FL): Psychological Assessment Resources.
30. Hendricks, C.M. (1996). Attention and memory training in childhood cancer survivors. *European Cancer Society Newsletter, 5*, 13–14.
31. How T. V., Hwang, A. S., Green, R. E. A. & Mihailidis, A. (2016): Envisioning future cognitive telerehabilitation technologies: a co-design process with clinicians. *Disability and Rehabilitation: Assistive Technology, 12(3)*, 244-261
32. Johnson, M. H., Halit, H., Grice, S. J., & Karmiloff-Smith, A. (2002). Neuroimaging of typical and atypical development: a perspective from multiple levels of analysis. *Developmental Psychopathology, 14(3)*: 521-536.
33. Kesler, S.R., Lacayo, N.J., & Jo, B. (2011). A pilot study of an online cognitive rehabilitation program for executive function skills in children with cancer-related brain injury. *Brain Injury, 25(1)*, 101-112.
34. Kueider, A. M., Parisi, J. M., Gross, A. L., & Rebok, G. W. (2012). Computerized Cognitive Training with Older Adults: A Systematic Review. *PLOS ONE 7(7)*: e40588.
35. Kurowski, B. G., Wade, S. L., Kirkwood, M. W., Brown, T. M., Stancin, T., & Taylor, H. G. (2013). Online problem-solving therapy for executive dysfunction after child traumatic brain injury. *Pediatrics, 132(1)*: e158-66.
36. Kurowski, B.G., Wade, S.L., Kirkwood, M.W., Brown, T.M., Stancin, T., & Taylor, H.G. (2014). Long-term benefits of an early online problem-solving intervention for executive dysfunction after traumatic brain injury in children: a randomized clinical trial. *JAMA Pediatric, 168(6)*, 523-531.
37. Laatsch, L., Harrington, D., Hotz, G., Marcantuono, J. Mozzoni, M.P., Walsh, V., & Hershey, K.P. (2007). An evidence-based review of cognitive and behavioral rehabilitation treatment studies in children with acquired brain injury. *Journal of Head Trauma and Rehabilitation, 22*, 248-256.
38. Levin, H.S., Ewing-Cobbs, L., & Eisenberg, H.M. (1995). Neurobehavioral outcome of pediatric head injury. In S. Broman, M. E. Michel (Eds.), *Traumatic Head Injury in Children* (pp. 70–94). Oxford: Oxford University Press.
39. Limond, J., Adlam, A. L. R., & Cormack, M. (2014). A model for pediatric neurocognitive interventions: considering the role of development and maturation in rehabilitation planning. *The Clinical Neuropsychologist, 28(2)*, 181-198.
40. Løhaugen, G. C., Beneventi, H., Andersen, G. L., Sundberg, C., Østgård, H. F., Bakkan, E., … Skranes, J. (2014). Do children with cerebral palsy benefit from computerized working memory training? Study protocol for a randomized controlled trial. *Trials, 15*: 269.
41. Lumos Labs (2010). *Lumosity: Reclaim Your Brain^TM^*. San Francisco, CA: Dakim, Inc.;. Available from: http://www.lumosity.com.
42. Malia, K., Law, P., Sidebottom, L., Bewick, K., Danziger, S., Schold-Davis E., … Vaidya, A. (2004). *The SCR Recommendations for Best Practice in Cognitive Rehabilitation Therapy.* Available at: http://www.societyforcognitiverehab.org/membership-and-certification/documents/EditedRecsBestPrac.pdf
43. Mostow, E. N., Walker, D., Frappaz, D., & Mulvihill, J. J. (1999). Quality of life in long-term survivors of CNS tumours of childhood and adolescence. *Journal of Clinical Oncology, 9,* 592-299.
44. Mulhern, R.K., & Palmer, S.L. (2003). Neurocognitive late effects in pediatric cancer. *Current Problems in Cancer, 27*, 177-197.
45. Ricker, J., Rosenthal, M., Garay, E., DeLuca, J., Germain, A., Abraham-Fuchs, K., Schmidt, K. U. (2002). Telerehabilitation needs: A survey of persons with acquired brain injury. *The Journal of Head Trauma Rehabilitation, 17(3),* 242-250.
46. Robertson, I. H., & Fitzpatrick, S. M. (2008). The future of cognitive neurorehabilitation. In D. T. Stuss, G. Winocur, I.H. Robertson (Eds.), *Cognitive Neurorehabilitation, Second Edition: Evidence and Application* (pp. 565-574). Cambridge: Cambridge University Press.
47. Schmeler, M. R., Schein, R. M., McCue, M. & Betz, K. (2009). Telerehabilitation Clinical and Vocational Applications for Assistive Technology: Research, Opportunities, and Challenges. *International Journal of Telerehabilitation, 1*, 12-24.
48. Sjö, N. M., Spellerberg, S. Weidner, S., & Kihlgren, M. (2010). Training of attention and memory deficits in children with acquired brain injury. Acta Paediatrica, 99(2), 230-236.
49. Slomine, B., & Locascio, G. (2009). Cognitive rehabilitation for children with acquired brain injury. *Developmental Disabilities Research Reviews, 15*, 133–143.
50. Souza, L. M., Braga, L. W., Filho, G. N., & Dellatolas, G. (2007). Quality-of-life: child and parent perspectives following severe traumatic brain injury. *Developmental Neurorehabilitation ,10*, 35–47
51. Spevack, T. (2007). A developmental approach to pediatric neuropsychological intervention. In S. J. Hunter & J. Donders (Eds.), *Pediatric neuropsychological interventions* (pp. 6-29). Cambridge: Cambridge University Press.
52. Tam, S. D., Man, W. K., Hui-Chan, C. W., Lau, A., Yip, B., & Cheung W (2003). Evaluating the efficacy of tele-cognitive rehabilitation for functional performance in three case studies. *Occupational Therapy International, 10*, 20-38.
53. Tavano, A., Grasso, R., Gagliardi, C., Triulzi, F., Bresolin, N., Fabbro F., & Borgatti, R. (2007). Disorders of cognitive and affective development in cerebellar malformations. *Brain, 10*, 2646-2660.
54. Taylor, H. G., Yeates, K. O., Wade, S. L., Drotar, D., Stancin, T., & Minich, N. (2002). A prospective study of short-and long-term outcomes after traumatic brain injury in children: Behavior and achievement. *Neuropsychology, 16(1),* 15–27.
55. Teasell, R., Bayona, N., Marshall, S., Cullen, N., Bayley, M., Chundamala, J., … Tu L. (2007). A systematic review of the rehabilitation of moderate to severe acquired brain injuries. *Brain Injury, 21(2)*, 107-112.
56. The WHOQOL Group. (1994). Development of the WHOQOL: Rationale and current status. *International Journal of Mental Health, 23 (3)*, 24-56. Available at: http://www.who.int/mental_health/publications/whoqol/en/
57. van‘t Hooft I, Andersson K, Bergman B, Sejersen T, von Wendt L, Bartfai A. Sustained favorable effects of cognitive training in children with acquired brain injuries. NeuroRehabilitation 2007; 22: 109–16.
58. van’t Hooft, I., Andersson, K., Sejersen, T., Bartfai, A., & von Wendt, L. (2003). Attention and memory training in children with acquired brain injuries. *Acta Paediatrica, 92*, 935–940.
59. Wade, S. L., Stancin, T., Kirkwood., M., Brown, T. M., McMullen, K. M., Taylor, H. G. (2014). Counselor-assisted problem solving (CAPS) improves behavioral outcomes in older adolescents with complicated mild to severe TBI. *Journal of Head Trauma and Rehabilitation, 29(3)*, 198-207.
60. Wade, S. L., Walz, N. C., Carey, J. C., & Williams, K. M. (2008). Preliminary efficacy of a Web-based family problem-solving treatment program for adolescents with traumatic brain injury. *Journal of Head Trauma and Rehabilitation, 23(6)*, 369-377.
61. Wechsler D. (2012). *Wechsler Intelligent Scale for Children – Fourth Edition (WISC-IV).* Italian Translation. Firenze: Organizzazioni Speciali.
62. Yeates, K.O. (2000). Pediatric closed-head injury. In K. O. Yeates, M. D. Ris, H. G. Taylor (Eds.), *Pediatric neuropsychology: Research, theory, and practice* (pp. 92–116). New York, NY: Guilford.
63. Zampolini, M., Todeschini, E., Guitart, M. B., Hermens, H., Ilsbroukx, S., Macellari, V., … Giacomozzi, C. (2008). Tele-rehabilitation: present and future. *Annali dell’Istituto Superiore di Sanità, 44(2)*, 125-134.
